# Electrochemical Synthesis of Sound: Hearing the Electrochemical Double Layer

**DOI:** 10.1021/acscentsci.3c01253

**Published:** 2024-02-20

**Authors:** Megan Kelly, Bill Yan, Christine Lucky, Marcel Schreier

**Affiliations:** † Department of Chemical and Biological Engineering, University of Wisconsin-Madison, Madison, Wisconsin 53706, United States; § Department of Chemistry, University of Wisconsin-Madison, Madison, Wisconsin 53706, United States

## Abstract

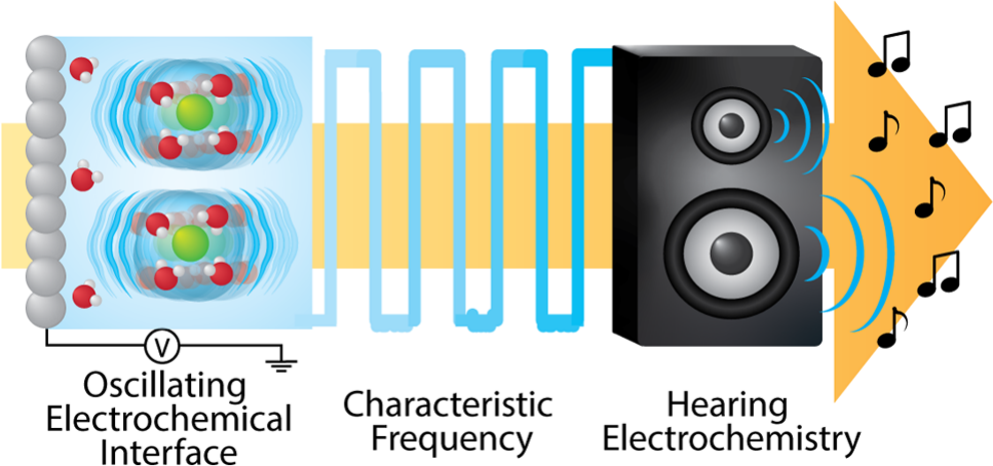

Electrochemical double layers (EDLs) govern the operation of batteries, fuel cells, electrochemical sensors, and electrolyzers. However, their invisible nature makes their properties and function difficult to conceptualize, creating an impediment to the broader understanding of double-layer function required for future technologies in energy storage and chemical synthesis. To render the behavior of electrochemical interfaces more intuitive, we made the rearrangement of interfacial components audible by employing the EDL as a variable element in a relaxation oscillator circuit. Connecting the circuit to a speaker generated an audible output corresponding to the change in potential resulting from EDL rearrangement. Variations in the applied voltage, electrolyte concentration and identity, as well as in the electrode material, yielded audible frequency variations that provide an intuitive understanding of EDL behavior. We expect that hearing the trends in behavior will provide a helpful and alternative method for understanding molecular movement at the electrochemical interface.

## Abstract 2

A technique to enable audible representation of the electrochemical double layer, giving intuitive insight into interfacial dynamics key to a sustainable future based on electrochemical technologies.

## Introduction

As efforts to combat climate change intensify, interest in electrochemistry for sustainable chemical synthesis and storage of renewable electricity has experienced enormous growth. At the heart of all electrochemical technologies is the electrochemical double layer (EDL), which is formed upon the contact of an electrode and an electrolyte. The electrostatic potential difference between the two phases, be it intrinsic or externally applied, leads to the movement of ions and solvent molecules at the electrode–electrolyte interface. This movement is due to the electrostatic and chemical forces between the electrode surface, electrolyte ions and solvent molecules.^1^ The resulting structure plays a key role in the mediation of electrochemical reactions. For example, the charge density in the EDL is known to control the production of ethylene in electrocatalytic CO_2_ reduction, and its structure has been shown to impact charge transfer rates in lithium-ion batteries and in photoelectrochemical cells.^2−7^

The invisible nature of the EDL makes its behavior challenging to probe and to understand. Existing methods to characterize the dynamics of ions at the EDL, such as electrochemical impedance spectroscopy (EIS), infrared (IR) and Raman spectroscopy, force-related techniques,^8−10^ X-ray spectroscopic methods, and electrokinetic streaming potentials,^11−16^ result in numerical data that are generally challenging to interpret. This creates an impediment to the intuitive understanding of interfacial properties. Human perception strongly relies on sounds. Technological systems, from computers to cars and crosswalk signals, leverage this fact by interacting with individuals through audible cues, which are intuitive to perceive. Audible signals also provide a more innate way to gain understanding of scientific principles.^17^ Indeed, data sonification has been used extensively in astronomy and is beginning to find applications in bioengineering to augment understanding of protein sequences.^18,19^ In another example, sound was found to be the most effective method to monitor the capture of CO molecules by scanning tunneling microscope tips.^20,21^

**Figure 1:**
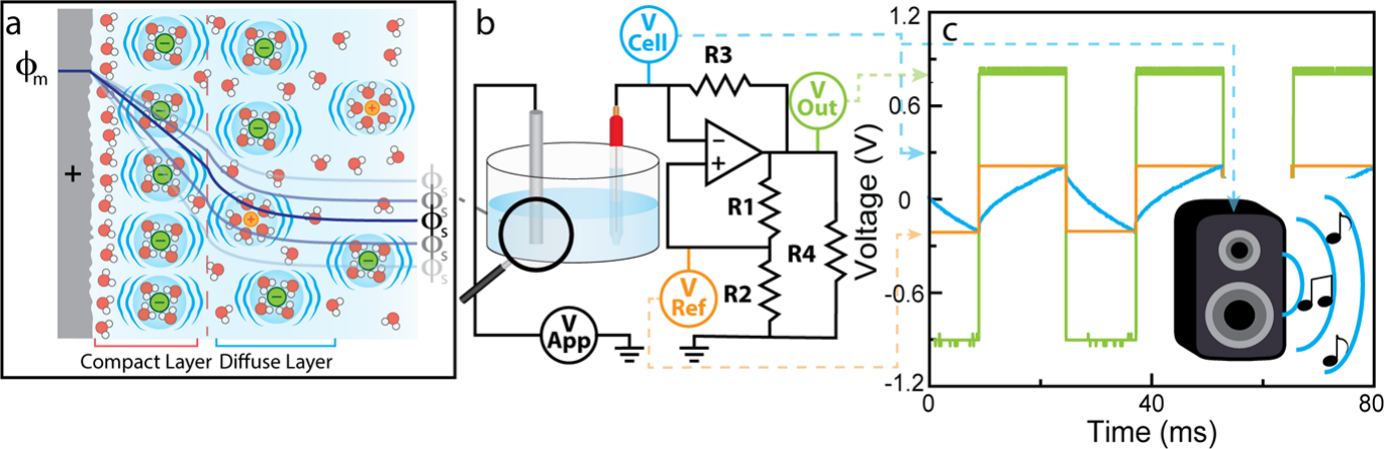
Overview of the electrochemical oscillator circuitry and function. (a) Visual depiction of the electrochemical interface and (b) its connection to the relaxation oscillator circuit. The fluctuation in solution potential from the circuit causes movement of ions, resulting in voltage waveforms (depicted with *V*_app_ = 0 V) (c) with a frequency specific to the electrochemical system at hand.

Inspired by the power of sound to express scientific phenomena, we herein sought a method to intuitively represent the molecular interactions occurring at EDLs by transforming the movement of the interfacial chemical constituents into audible frequencies. To realize this vision, we initiated oscillation of an electrochemical interface using a relaxation oscillator circuit. The oscillation of the interface generates voltage waveforms which are converted to sound by connecting the electrochemical cell to a speaker, allowing EDL rearrangements to be tracked audibly and instantaneously (Figure 1). At the molecular level, the audio signal directly represents the movement of ions and solvent molecules in the double-layer. Therefore, the output frequency of the circuit is dependent on a multitude of EDL properties, including the applied potential, solvent identity, electrolyte concentration, electrolyte identity, and electrode material. Because the output frequency is specific to the identity of the system, it reflects the molecular interactions occurring at the EDL. Using our approach, we are able to listen to changes in pitch induced by modifications to the concentration and identity of electrolyte ions, the applied potential across the electrochemical interface, the electrode identity, and the adsorption of compounds to electrode surfaces. Our findings show that sound is a powerful medium for monitoring EDL behavior while also opening new avenues for bridging art and science.

## Results and Discussion

To probe the properties of the EDL using sound, we integrated an electrochemical cell into a relaxation oscillator circuit (Figure 1). The function of a relaxation oscillator is to generate a specific output frequency by continuously charging and discharging a capacitor through a feedback resistor (R3, Figure 1b). Altering the properties of the capacitor results in a change in the output frequency, due to the inverse relationship between frequency and capacitance (Figure S2, eqs S1 and S2). In our setup, we replace the capacitor with a two-electrode electrochemical cell, consisting of the working electrode (WE) and an Ag/AgCl (3 M KCl) counter electrode (equivalent circuit available in Figure S3). As the circuit oscillates, the potential difference across the cell fluctuates at a magnitude controlled by resistors R1 and R2, with the measured voltage depending on the configuration of the working electrode EDL. The resulting frequency of oscillation is a function of the time it takes to charge and discharge the WE interface via the dynamic rearrangement of ions and solvent molecules. This time, which is related to the interfacial capacitance, depends on the identity of the electrode, the electrolyte, and the applied potential. Interfaces that store more charge have a higher capacitance, and thus take longer to rearrange, resulting in a lower frequency. The inverse is true for interfaces storing small amounts of charge. Since the relevant oscillation frequencies are in the audible range, our setup allowed us to listen to the rearrangement of electrolyte components and intuitively monitor the behavior of the EDL as we probed the impact of changing interfacial components. This was made possible by connecting an audio amplifier directly to the oscillating electrochemical cell (Figure 1) and recording the generated sounds, which are made available in Videos S1–S6. A detailed discussion of the oscillation cycle is available in the Supporting Information and depicted in Figures S5–S9. Our approach deviates from techniques such as electrochemical impedance spectroscopy (EIS) in both how the oscillations are induced and how data is extracted from the system. In EIS, a range of known frequencies of AC voltage are applied to the interface, and the resulting current amplitude and phase shift are used to gain insight into electrochemical processes. In contrast, our approach uses the frequency of the potential oscillation as an output to acquire an understanding of interfacial behavior and properties. Our system provides a simple and fast method to screen interfacial properties and gain an intuitive sense of interfacial behavior.

### Impact of Applied Voltage on Audible Signal

The applied potential is a crucial handle for tuning electrocatalytic reactions, as changes in electrochemical reaction rates result from the modification of the reaction microenvironment via the potential-induced rearrangement of charged compounds.^22−24^ By listening to the variation in pitch as the potential changed (Video S1), we gained intuitive insight into the potential regions where significant changes in EDL behavior occurred for a polycrystalline Pt electrode immersed in 7 mM KClO_4_.

**Figure 2:**
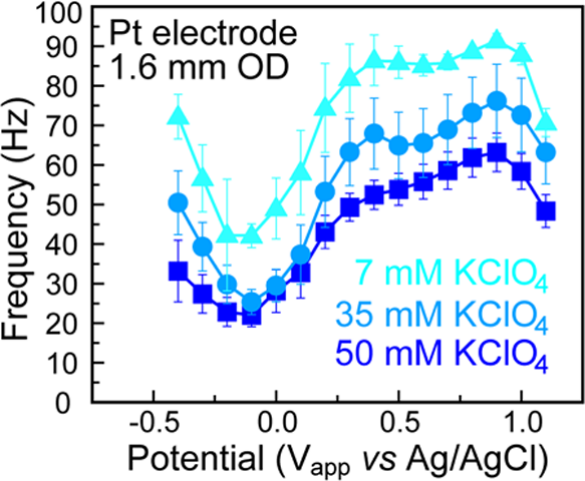
Frequency vs applied potential data for 7, 35, and 50 mM solutions of KClO_4_ on a Pt electrode. Error bars represent one standard deviation above and below the mean computed from three trials. OD = outer diameter.

As we increased the voltage from −0.4 to 1.1 V (Video S1, Figure 2), we observed a complex trend of first decreasing pitch, followed by a rapid increase in pitch past −0.1 V, an extended plateau of constant pitch between 0.4 and 0.9 V, and finally again a steep decrease in pitch above 1.0 V. Clearly, an electrochemical cell is not a “well-tempered” musical instrument.^25^ Yet, the non-monotonous potential-dependence exhibited by the observed pitch is directly related to a series of complex electrochemical dynamics occurring at the Pt-electrolyte interface, some of which have previously been described. For example, a distinct minimum in pitch was observed at −0.1 V, which corresponds to a maximum in interfacial capacitance due to the inverse relationship between frequency and capacitance.

Such a capacitance maximum has been explained by Pajkossy and Kolb, who suggested that Pt exhibited a potential-dependent capacitance peak where water molecules at the electrode surface flip their dipoles from a “hydrogen down” to an “oxygen down” configuration, thus inverting the way they interact with the interfacial electric field.^26,27^ In addition to this minimum likely caused by water reorientation, the distinct plateau in pitch between 0.4 and 0.9 V relates to a similar plateau seen in capacitance measurements that was attributed to an interplay between impurities and Pt–O bond formation.^28^ Finally, the strong decrease in pitch observed at potentials greater than 0.9 V can likely be explained by the formation of bulk platinum oxide which has a high dielectric constant, thus increasing the system’s ability to store charge, and thereby the time required to rearrange the interface.^28^

**Figure 3:**
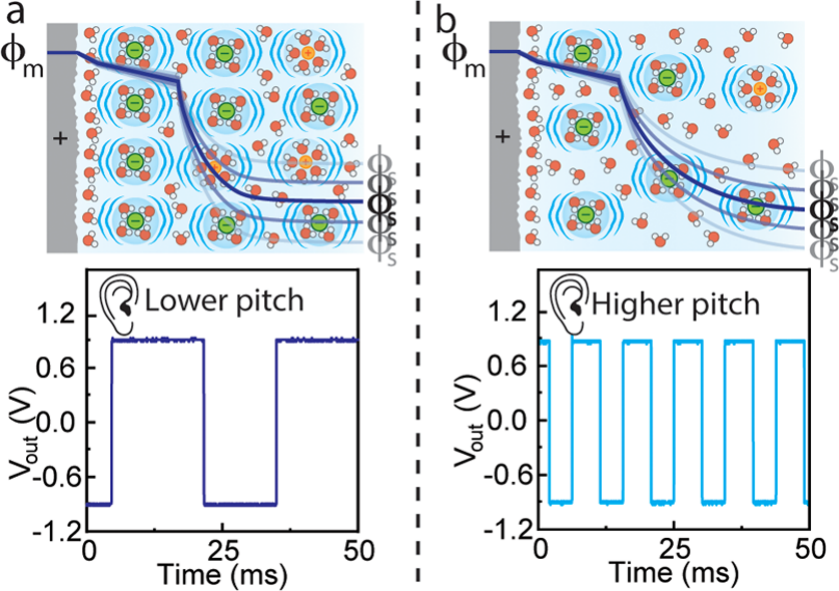
Visual representation of the electrochemical interface at (a) high and (b) low electrolyte concentrations. The increased number of charges present in (a) takes longer to move than the smaller amount of charge in (b), resulting in a lower pitch. Illustrations not to scale.

This time can also be modulated by the electrolyte concentration. Indeed, increasing the electrolyte concentration from 7 to 50 mM KClO_4_ led to a decrease in pitch at all potentials. This result is expected, because at higher electrolyte concentrations, more ions are recruited to the interface under a constant potential drop (Figure 3), thus increasing the time it takes for interface to rearrange. Despite the decrease in frequency due to the increase in concentration, the complex, nonmonotonic trends in frequencies across different potentials were maintained across the three electrolyte concentrations. This complex trend was also preserved regardless of the magnitude of oscillation applied (Figure S15), further demonstrating how the sounds generated by changing the potential across a simple Pt-electrolyte interface reveal complex molecular dynamics. In the following, we discuss how this complexity is further magnified as the interface is changed via electrolyte modifications.

### The Role of Cation Identity in Determining Pitch

Fascinating trends are observed when considering the impact of electrolyte cations on the sounds generated by the Pt-electrolyte interface. It is well-known that the identity of the electrolyte ions can tune the outcome of electrochemical processes. This is particularly prominent in electrocatalytic CO_2_ and CO reduction, where cation identity controls the rate of methane vs ethylene production,^6^ and quaternary alkylammonium salts have been found to decrease ethylene formation.^29^ These phenomena have been related to the magnitude of the interfacial potential drop, which at high electrolyte concentrations depends on the identity of electrolyte cations, as well as to the ability of hydrophobic cations, such as alkylammonium compounds, to displace interfacial water and thereby modulate electrochemical reactivity. These phenomena also occur in the context of electrochemical sounds. The EDL capacitance is strongly affected by the proximity of the outer Helmholtz plane to the electrode surface. Therefore, ions with larger solvated radii are expected to decrease the interfacial capacitance, which would increase the audible pitch.^1^ To probe the impact of cation nature on the sound generated by interfacial rearrangement, we compared Pt electrodes in 1 M solutions of Et_4_NCl, Pr_4_NCl, and Bu_4_NCl, which each feature distinct size and hydrophobicity.

**Figure 4:**
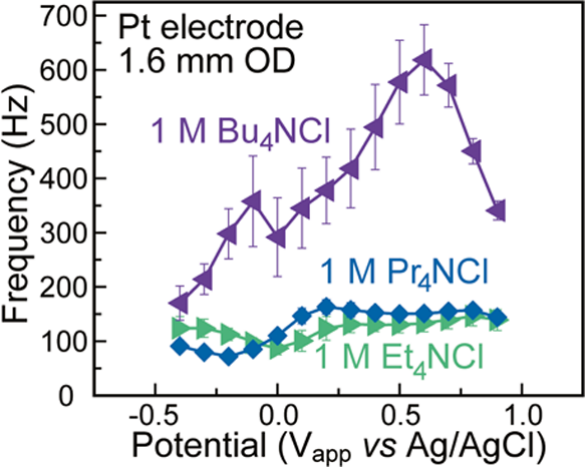
Frequency vs applied potential data for 1 M solutions of Et_4_NCl, Pr_4_NCl, and Bu_4_NCl electrolyte on a Pt electrode. Error bars represent one standard deviation above and below the mean computed from three trials.

The sounds generated by the three alkylammonium salts demonstrated the strong impact cation identity has on EDL behavior (Figure 4). As shown in Video S2, at potentials greater than −0.1 V, Pr_4_NCl generated a higher pitch than Et_4_NCl, and the pitches gradually converged as the potential became more oxidative. This is expected, as Pr_4_NCl has a larger cationic radius,^30^ and chloride likely begins to dominate the interface at highly oxidative potentials. Interestingly, at potentials more reductive than −0.1 V, Et_4_NCl switched to producing a higher pitch than Pr_4_NCl, revealing behavior that can be a topic of future studies.

**Figure 5:**
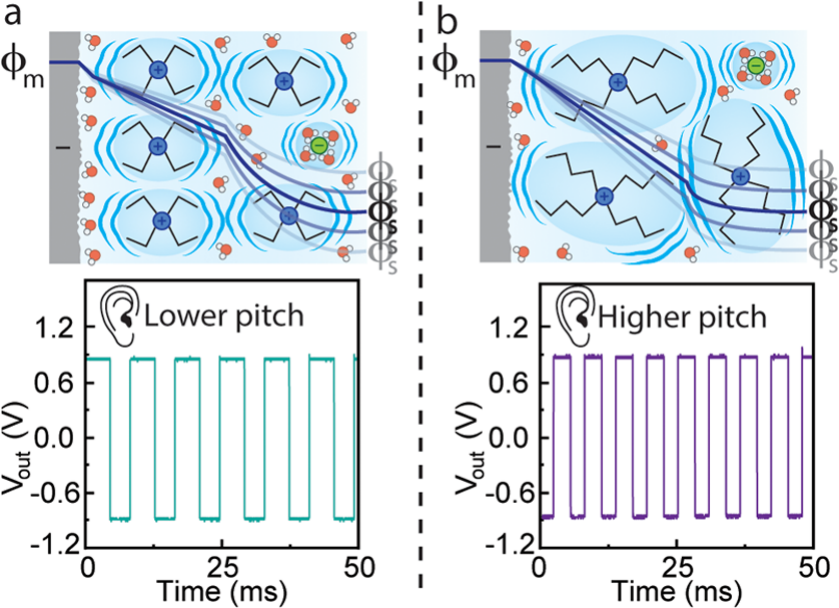
Visual representation of the electrochemical interface in a (a) Et_4_NCl and (b) Bu_4_NCl electrolyte. Et_4_N^+^ is smaller than Bu_4_N^+^. Additionally, we theorize that Bu_4_N^+^ disrupts interfacial water more than Et_4_N^+^, leading to a decrease in capacitance and thus a significant increase in pitch. Illustrations not to scale.

The complexities of interfacial interactions are again highlighted by the dramatic difference in sounds generated in the presence of Bu_4_NCl relative to the other tested alkylammonium electrolytes. This difference can be heard in Video S2. Bu_4_NCl generated a pitch that had at least twice the frequency than that observed for Et_4_NCl and Pr_4_NCl. An increase was expected due to the increased cationic radius of Bu_4_N^+^; however, the increase in solvation radius from Et_4_N^+^ to Pr_4_N^+^ is larger than that between Pr_4_N^+^ and Bu_4_N^+^,^30^ indicating another effect is contributing to the change in pitch. We hypothesize that the overall increase in pitch with Bu_4_NCl results from the hydrophobicity of the Bu_4_N^+^ ion and its tendency to displace water from the electrode surface (Figure 5). Indeed, the Bu_4_N^+^ cation has been suggested to form films on electrode surfaces, which will lead to a significant decrease in capacitance due to the substantially lower dielectric constant of Bu_4_N^+^ compared to H_2_O,^31^ decreasing the amount of charge that can be stored at the interface. The combination of surface water displacement and possible film formation are therefore likely behind the sharp increase in pitch between 0 and 0.6 V. Positive of 0.6 V, we hypothesize that the Bu_4_N^+^ cations near the electrode surface are displaced by Cl^–^ and H_2_O, yielding a drastic decrease in pitch that approaches the case for the other tested alkylammonium electrolytes.^32^ We attribute the decreasing pitch negative of −0.1 V to increased charge packing and hydrogen deposition at the interface which also decreases the impact of water displacement by Bu_4_N^+^.^32^

### Observation of Interfacial Cation Competition

Having the ability to listen to the sounds generated by the molecular interactions at electrochemical interfaces provides an avenue to hear how the interface reacts to real-time changes in electrolyte properties. Interest is growing in surface modifiers that displace interfacial water to tune the product selectivity of CO_2_ reduction, a reaction that is most commonly carried out in electrolytes containing alkali cations.^29,33,34^

We therefore expected that introducing hydrophobic modifiers, such as Bu_4_N^+^ described above, into the electrolyte, would allow us to listen in real time to the interfacial change as water is displaced from the electrode surface. Consequently, we recorded the sound generated when adding 2 mL of 1 M Bu_4_NCl into 10 mL of 1 M NaCl electrolyte with the Pt electrode biased at 0 V vs Ag/AgCl. Surprisingly, as shown in Video S3, no meaningful change in sound was heard, indicating that the interfacial properties remained largely unchanged upon introducing Bu_4_N^+^. However, when we poured 2 mL of 1 M NaCl into 10 mL of 1 M Bu_4_NCl electrolyte, we observed an instantaneous and strong decrease in pitch as can be heard in Video S4. This indicated that upon their addition, Na^+^ ions started to dominate the interface and were able to displace Bu_4_N^+^, transforming the interface into a configuration that is more reflective of the one formed in the sole presence of a Na^+^-based electrolyte. The ability to listen to changes in the EDL in real-time provides an opportunity to use sound as a fast-screening method to gain intuitive understanding of mixed electrolyte systems. Hearing the sounds generated during the process of modifying the properties of the electrolyte thus allowed us to gain direct and intuitive insight into how electrolyte ions compete at interfaces, shedding light on the important interaction of hydrophilic and hydrophobic ions at Pt electrodes.

### Impact of Anion Adsorption on Audible Signal

In addition to displacing water from the electrochemical interface, electrolyte ions can specifically adsorb onto the electrode surface, causing changes in the EDL structure by blocking adsorption sites on the electrode.^35^ In electrocatalysis, it is known that the adsorption of chloride ions on a Pt electrode will inhibit the formation of platinum oxide and thus suppress the oxygen reduction reaction.^36−38^

**Figure 6:**
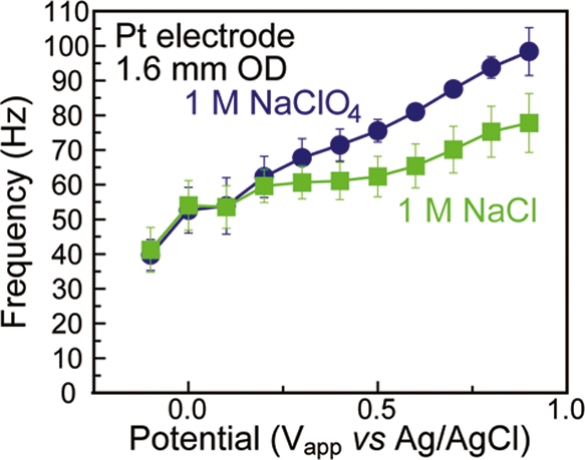
Frequency vs applied potential data for 1 M solutions of NaClO_4_ and NaCl electrolyte on a Pt electrode. Error bars represent one standard deviation above and below the mean computed from three trials.

Having observed that forming platinum oxide caused an increase in audible pitch of the interface (see above), we hypothesized that introducing chloride ions would cause a decrease in audible frequency due to a suppression of surface oxide formation relative to ClO_4_^–^ anions, which do not adsorb onto the electrode surface.^5,35,39^ Indeed, as can be heard in Video S5, adding 2 mL of 1 M NaCl into 10 mL of 1 M NaClO_4_ at 0.6 V vs Ag/AgCl resulted in an instantaneous decrease in pitch, potentially reflecting chloride adsorption that minimizes the formation of surface oxide compounds.^5,35,39^ Additionally, a distinct voltage-dependent change in pitch could be heard due to the presence of Cl^–^. Relative to ClO_4_^–^, Cl^–^ ions led to a decrease in pitch at the positive end of the tested potential range. However, the Cl^–^ ions had minimal impact at potentials negative of 0.3 V, a potential which was within the range of previously reported potentials of zero charge (PZCs) on polycrystalline Pt (Figure 6).^40,41^ The absence of changes at potentials negative of the apparent PZC was expected, as the interface should be dominated by water and electrolyte cations, in this case Na^+^, whose identity and concentration remained unchanged.^1^ The ability to hear chloride adsorption, both instantaneously and in trends across voltages creates an opportunity to use sound to probe interactions between anions and the electrode surface.

### Impact of Electrode Identity on Audible Signal

Electrochemical interfaces emerge from the chemical and electrostatic interaction between the electrolyte and the electrode material. Thus far, we have discussed a plethora of interfacial phenomena that occur, such as interactions between the electrode material, the solvent, anions, cations, as well as water molecules and chemical adsorbates from the viewpoint of the electrolyte. These interactions influence the position and orientation of interfacial ions and dipolar species, which in turn determine the magnitude of the potential drop at the electrode.^1^ However, the potential profile resulting from these interactions is also strongly impacted by the electrode material, as the potential profile additionally depends on the difference in work function between the electrode and the electrolyte.^42^ To study the impact of the electrode material on EDL behavior, we recorded the sounds generated by Cu, Ti, and Pt electrodes in 80 mM KClO_4_ electrolyte. We observed substantial changes to the sound produced by rearrangements at the EDL when comparing the different electrodes, which emphasized the key role the electrode material plays in determining the behavior of electrochemical interfaces.

**Figure 7:**
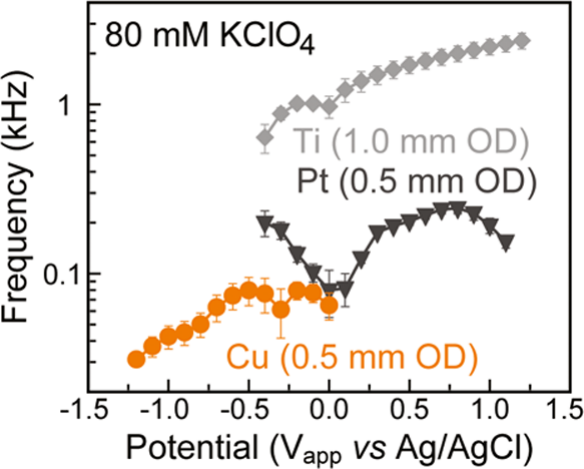
Comparison of frequencies between Cu, Ti, and Pt electrodes in 80 mM KClO_4_. Error bars represent 1 standard deviation above and below the mean computed from three trials.

Copper and titanium both showed trends in pitch that could be explained by previous literature. The highest pitch generated by copper occurred at −0.2 V and −0.5 V (Figure 7), roughly corresponding to the local capacitance minima observed on Cu in 0.2 M NaClO_4_ electrolyte.^43^ These capacitance minima were hypothesized to be related to the formation of Cu–H compounds prior to hydrogen evolution.^43^ Titanium demonstrated a continuous increase in frequency from 0 to 1.2 V, which is consistent with literature reports of a continuous decrease in capacitance due to the formation of TiO_2_ on the electrode surface.^44^ The range of frequencies spanned by these materials in the same electrolyte gives a sense of the vast differences in their interactions with electrolyte constituents and demonstrates the need for continued studies into interface function. It is important to note that Ti provides the most linear response in pitch with applied voltage, which makes it an appropriate electrode material for designing an “electrochemical keyboard” that varies the applied voltage to control the pitch generated by depressed keys.

### Electrochemical Keyboard

While we initially designed our electrochemical oscillation circuit as a means to gain deeper understanding of the complex dynamics occurring at electrochemical interfaces, as a proof of concept, we also designed a musical keyboard based on electrochemical oscillation. Inspired by the voltage-controlled oscillators (VCOs) designed for synthesizing electronic music,^45^ we used the control voltage output of an electronic keyboard to modulate the potential applied across an electrochemical interface. This allowed us to design a keyboard-controlled VCO that uses the electrochemical cell as the tunable element. In electronic music, the control voltage output of keyboards is defined so that a one volt difference in potential corresponds to changing the pitch by one octave, which means doubling the frequency. This roughly mirrors the frequency response of the Ti-electrolyte interface, allowing us to design a musical instrument, an “electrochemical synthesizer”, that can be played like a conventional keyboard (Figure S14, Video S6).

## Conclusion

In this work we demonstrate that the characteristics of electrochemical interfaces can be transformed into sounds by bringing the EDL into oscillation. Using an electrochemical cell as the tunable element in a relaxation oscillator circuit allows for changes in the EDL structure to be transformed into audible signals. Varying the applied potential, the electrolyte concentration, cation identity, anion identity and the electrode material strongly influenced the sound produced. Additionally, interfacial rearrangements due to cation competition and anion adsorption could be heard in real-time, enabling an intuitive manner to study dynamic EDL rearrangements. The auditory signal generated by oscillating electrochemical interfaces provides an unconventional avenue to interpret the abstract molecular phenomena characterizing interface behavior.

Understanding the molecular dynamics of electrified interfaces is essential for innovations in energy storage and sustainable chemical synthesis. We expect the insight gained in this work to shed light on underappreciated interactions characterizing interface function, which are critical to researchers active in these areas of electrocatalysis. We further foresee our approach as a tool that can open new opportunities for artistic expression at the interface between science and electronic music.

## Materials and Methods

### Electrolyte Preparation

Potassium perchlorate (≥99%, Sigma-Aldrich), sodium perchlorate (98–102% Alfa Aesar), sodium chloride (99.85%, Fisher Scientific), tetraethylammonium chloride (99%, Thermo Scientific), tetra-n-propylammonium chloride (99%+, Thermo Scientific), and tetrabutylammonium chloride (98%, AstaTech) were used as-is to prepare electrolytes with water supplied from a Milli-A Integral Water Purification System (EMD Millipore).

### Electrode Material Preparation

Cu (99.9%, Craft Wire), 0.5 mm diameter Pt (99.99%, Kurt J. Lesker, 0.5 mm diameter), and Ti wires (≥99.99%, Fisher Scientific, 1.0 mm diameter) were immobilized in a 1 mL polypropylene syringe with epoxy and allowed to set overnight. The immobilized wires were polished with a successive series of sandpaper (1200 then 2500 grit), 1 μm alumina and 0.3-μm alumina. In between polishings, the electrodes were sonicated in Milli-Q water for 5 min. PTFE plumbing tape was stretched over the exposed epoxy to provide an inert barrier during electrochemical experiments. Electrodes were rinsed with Milli-Q water and patted dry with a KimWipe prior to use in experiments. Electrodes were stored in centrifuge tubes under atmospheric conditions between experiments.

For the concentration, cation, and anion studies, a BASi standard working platinum electrode (Pt) - 1.6 mm diameter, 99.95% purity was used. The electrode was rinsed with Milli-Q water prior to use and stored under atmospheric conditions in the manufacturer container with the provided end-cap attached. The electrode surface was polished with 0.3-μm alumina suspension and sonicated for 5 min in Milli-Q water prior to its use in experimentation.

### Circuit Function

The circuit functions by using an operational amplifier (op-amp) to compare input voltages at its inverting (labeled '–') and noninverting (labeled ‘+’) input. The noninverting input is set to a reference voltage dictated by the type of op-amp and the values of resistors R1 (33 kΩ) and R2 (33 kΩ). The inverting input is connected to a feedback resistor R3 (10kΩ) and a capacitor. When the circuit is powered, the capacitor will begin charging toward the positive output saturation voltage. However, when the voltage at the inverting input reaches the reference voltage at the noninverting input, the sign of the output voltage is reversed. This causes the capacitor to begin discharging toward the negative saturation voltage. The change in sign of the output voltage also changes the sign of the reference voltage, thus the output voltage will reverse again once the inverting input voltage reaches the noninverting input voltage.^46^ The circuit does not moderate the gain of the op-amp, which is therefore operating in a saturated state. This causes the output voltage at the op-amp to have a square waveform, which is alternating between the maximum and minimum supply voltage of the op-amp.

### Frequency Collection

A Keithley 2230-30-1 triple channel DC power supply was used to power the op-amp of the circuit and provide DC power to the electrochemical cell. Channels 1 and 2 were linked together to create a bipolar power supply for the op-amp, while channel 3 was connected to the working electrode in the cell. A BASi Ag/AgCl (3M) reference electrode was used as the counter electrode, and this was connected to the inverting input of the op-amp. The capacitance of the reference electrode was independent of frequency over the ranges relevant to this study, indicating it behaved purely as a resistor, thus the capacitance was dominated by the capacitance at the working interface. A Tektronix TBS 1052B-EDU oscilloscope collected the output frequency data and plotted waveforms. The oscilloscope and power supply were controlled via a LabView program which changed the applied potential to the cell and recorded frequency vs time and potential. Each potential was held for 120 s. Anodic sweeps starting at 0 V were conducted first and swept until 1.1 V (concentration studies) or 0.9 V (cation and anion studies) vs Ag/AgCl in 0.1 V increments. The system was returned to 0 V and reconditioned before the cathodic sweeps were started. Anodic and cathodic sweeps were performed separately due to the need to manually switch the cables on the power supply to provide a negative voltage. Potential ranges for the sweeps were determined by the limits of applied voltage at which the system stopped oscillating.

### Solution Resistance Compensation

For the dilute concentration studies, to prevent any convolution of frequency increase with increased solution resistance, an external decade box resistor was placed between the reference electrode and its connection to the circuit (Figure S10). The total resistance of the decade box and solution resistance was set to 5.2 kΩ for all runs. A hybrid EIS measurement was performed before each anodic and cathodic sweep to measure the series resistance and adjust the decade box accordingly. The materials study did not require this adjustment as the electrolyte concentration was constant across all materials. For the 1 M studies, all solution resistances measured with EIS were less than 200 Ω, which was less than 2% of the 10 kΩ resistance of R3 and thus deemed to be negligible.

### Electrode Conditioning

Prior to any frequency collection, electrodes were conditioned using a Gamry Reference 600 Potentiostat in a 3-electrode setup, the counter electrode was a 0.5 mm diameter Pt wire submerged at least 2 cm into solution. The reference electrode was a 3 M KCl Ag/AgCl electrode from BASi. The conditioning procedures were as follows for the various materials:

Platinum used a cyclic voltammogram (CV) from −0.216 V to 1.1 V vs *E*_ref_, with the initial V at 0.1 V vs *E*_ref_ and the final V at 0.1 V vs *E*_ref_ with a scan rate of 100 mV/s for 10 cycles. Titanium used chronoamperometry (CA) for 240 s at −1.5 V vs *E*_ref_, and copper used CA for 180 s at −1.2 V vs *E*_ref._

Electrodes were conditioned in the experimental solution for the concentration and electrode materials studies. For sweeps conducted at 1 M concentrations, the electrodes were conditioned in 7 mM KClO_4_ to prevent any surface roughening from the concentrated salts prior to frequency collection. The electrodes were rinsed in Millli-Q water and dried prior to being used in the experimental setup. Electrodes were reconditioned between anodic and cathodic sweeps. Conditioning procedures were developed to sufficiently clean the electrode surface such that the frequency at 0 V was not dependent on whether the prior sweep was cathodic or anodic.

### Data Processing

The LabView program output an Excel spreadsheet denoting the time spent at each potential, the measured frequency and the applied potential. A MATLAB code was used to convert the individual time spent at each potential into total experimental time. Average frequencies per potential were determined by averaging the frequencies during the last 60 s of the potential hold, which allowed for 60 s of system stabilization.

## Supporting Information Available

Detailed information on circuit components; relationship between capacitance and frequency; equivalent circuit of electrochemical cell; detailed explanation of oscillation cycle; photographs of the setup; impact of oscillation amplitude on frequency trends; videos of the sounds generated by the system (PDF)
Video S1, comparison of audio for 7 mM and 50 mM KClO_4_ between −0.4 and 1.1 V (MP4)
Video S2, comparison of audio for 1 M Bu_4_NCl, Et_4_NCl, and Pr_4_NCl between −0.4 and 1.1 V (MP4)
Video S3, introduction of 1 M NaCl into 1 M Bu_4_NCl at 0 V (MP4)
Video S4, introduction of 1 M Bu_4_NCl into 1 M NaCl at 0 V (MP4)
Video S5, introduction of 1 M NaCl into 1 M NaClO_4_ at 0.6 V (MP4)
Video S6, video of “electrochemical synthesizer” using a Ti electrode in 80 mM KClO_4_ (MP4)

## Abbreviations

PZC: Potential of zero charge
VCO: Voltage-controlled oscillator
EDL: electrochemical double layer
EIS: electrochemical impedance spectroscopy
XPS: X-ray photoelectron spectroscopy
SAXS: small-angle X-ray scattering
XAS: X-ray absorption spectroscopy
CV: cyclic voltammetry
CA: chronoamperometry
