# Supplementary material for: Electrochemical Synthesis of Sound: Hearing the Electrochemical Double Layer
Source: ACS Cent Sci. 2024 Feb 20;10(3):595–602. doi: 10.1021/acscentsci.3c01253 (PMC10979475; doi:10.1021/acscentsci.3c01253)
Supplement: Supplementary file 1 — oc3c01253_si_001.pdf [file oc3c01253_si_001.pdf]

Supporting Information:

Electrochemical Synthesis of Sound: Hearing the Electrochemical Double Layer

*Megan Kelly[a], Bill Yan[a], Christine Lucky[a], Marcel Schreier[a,b] **

[a] Department of Chemical and Biological Engineering, University of Wisconsin−Madison, Madison, Wisconsin 53706, United States;

[b] Department of Chemistry, University of Wisconsin−Madison, Madison, Wisconsin 53706, United States;

E-mail: mschreier2@wisc.edu

Supporting Information Contents

- Detailed Information on Circuit Components
- Relationship Between Capacitance and Frequency
- Equivalent Circuit of Electrochemical Cell
- Detailed Explanation of Oscillation Cycle
- Photographs of the Setup
- Impact of Oscillation Amplitude on Frequency Trends
- Figures S1 to S15
- Table S1
- Legends for Movies S1 to S6
- References

Other supporting materials for this manuscript include the following:

- Video S1 to S6

Detailed Information on Circuit Components

In the following we detail the circuit layout and the employed components.

R1: 33 kΩ resistor (low concentration)**,** 100k resistor (1 M runs)
R2: 33 kΩ resistor
R3: 10 kΩ resistor
R4:, 23 Ω resistor
Op-amp: OPA551PA (Texas Instruments)

All voltages in the main text are plotted centered on 0 V for clarity. Due to a small asymmetry in the power supply, at pin 2 there is a −0.04 V DC offset, and at pin 6 there is a −0.1 V DC offset. The magnitudes of the voltages plotted are corrected for the offset to reflect the true voltage magnitude spanned. We verified that this did not impact the measured frequency.

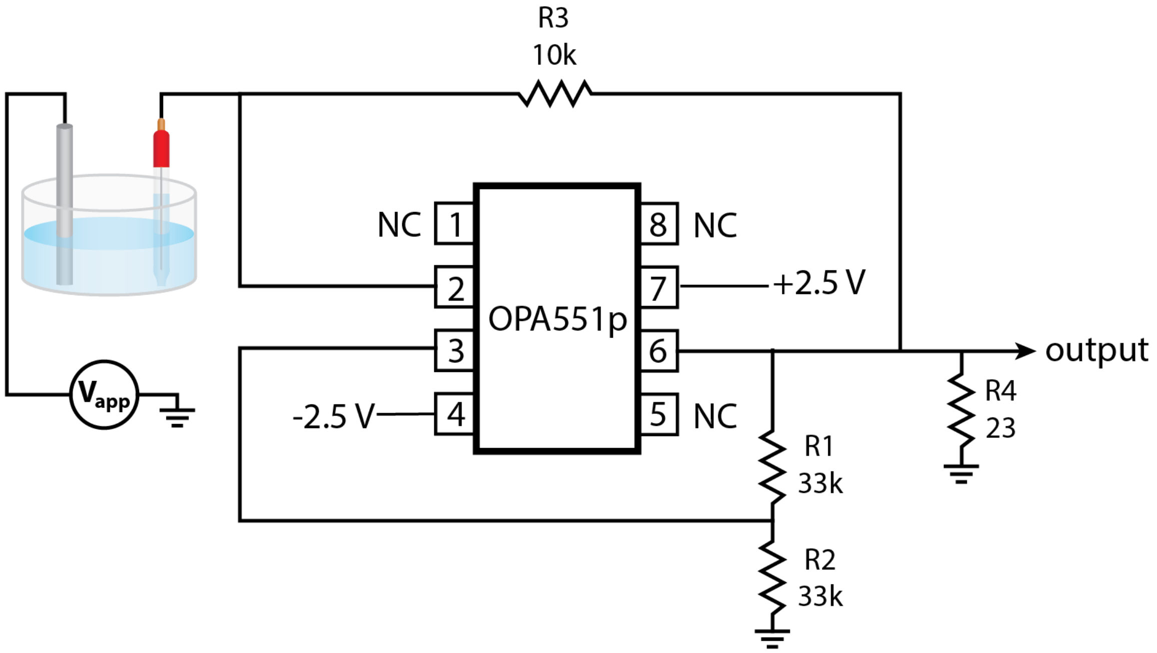

Figure S1. Detailed circuit diagram with op-amp pinout. R1 depicted as set for runs with concentrations <1 M. Runs with a concentration of 1 M had an R1 value of 100k.

Relationship Between Capacitance and Frequency

As shown in **Equation S1** and **S2** the output frequency of a relaxation oscillator circuit is linearly proportional to the inverse of the capacitance, (**Equation S1, S2)**.

$$f = \frac{1}{2R_3C*\ln\frac{1+\beta}{1-\beta}} \propto \frac{1}{C} \quad \textbf{(S1)}$$

$$\beta = \frac{R_2}{R_1+R_2} \quad \textbf{(S2)}$$

In these equations, f is the output frequency of the circuit, R_3 is the resistance of the feedback resistor R3, C is the capacitance of the circuit capacitor, and β is the ratio of resistances between R1 and R2 as described in **Eq. S2**. We have verified this relationship experimentally (**Figure S2**) using capacitors. If additional elements of the electrochemical interface such as series and charge transfer resistances become relevant in magnitude, numerical simulation is required to devise oscillation frequencies.

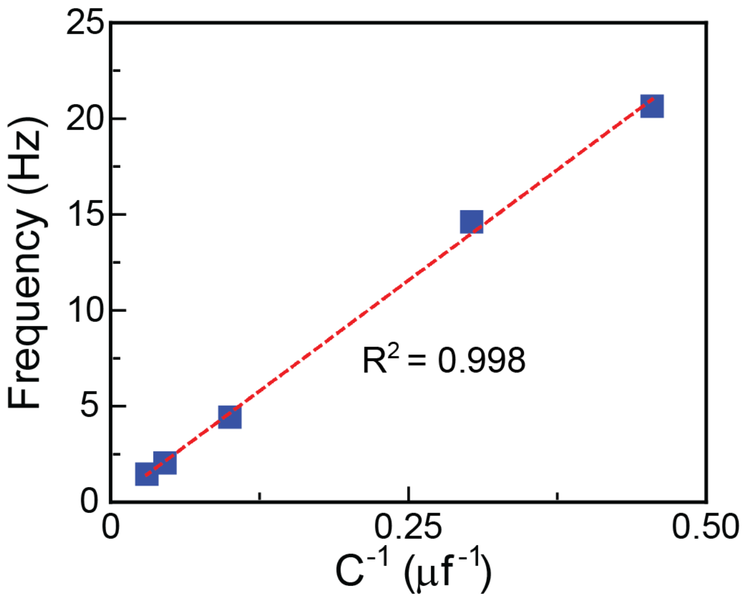

Figure S2. Linear fit of frequency versus inverse capacitance, as is consistent with expected circuit behavior.

Equivalent Circuit of the Electrochemical Cell

As shown below in **Figure S3**, the equivalent circuit for our two-electrode electrochemical cell is modeled as two Randles circuits in series connected through the series resistance. Utilizing an Ag/AgCl (3 M KCl) reference electrode as the counter electrode enabled us to probe the working electrode interface in isolation as a good reference electrode acts as an ideal resistor over a wide frequency range. We verified this using electrochemical impedance spectroscopy, which showed that the phase angle of the reference electrode is near-zero at all frequencies relevant to our system (**Figure S4**).

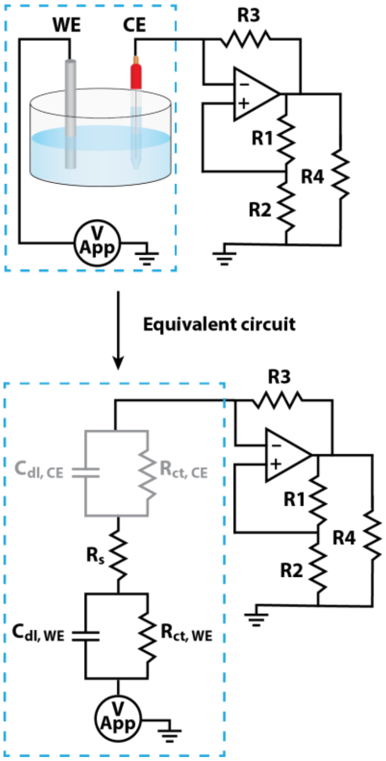

Figure S3. Circuit diagram with equivalent circuit for 2-electrode electrochemical cell depicted with 2 simplified Randles circuits in series. $C_{dl,CE}$ = double layer capacitance for the counter electrode. $R_{ct,\ CE}$ = charge transfer resistance for the counter electrode. R_s = series resistance. $C_{dl,WE}$ = double layer capacitance of the working electrode. $R_{ct,WE}$ = charge transfer resistance of the working electrode.

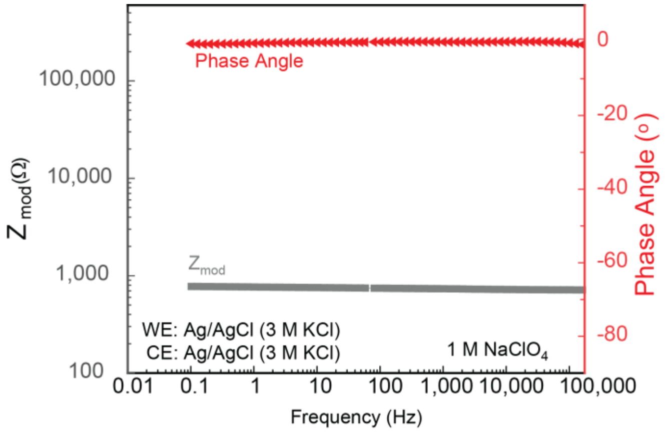

Figure S4. Electrochemical impedance spectroscopy of Ag/AgCl (3 M KCl) reference electrodes. This data was recorded using a two-electrode cell where both the working and counter electrodes are 3 M KCl Ag/AgCl electrodes, demonstrating resistive behavior over the frequency range relevant to this study.

Detailed Explanation of Oscillation Cycle

A relaxation oscillator circuit generates a square wave voltage output with a specific frequency based on the time constant obtained by continuously charging and discharging a capacitor through a feedback resistor. The circuit generates a square wave output because the operational amplifier (triangle symbol in **Figure S3**) is operating at saturation, meaning it switches between outputting its maximum positive and negative supply voltages. In our circuit, the capacitor is replaced by a two-electrode electrochemical cell (**Figure S1**), dominated by the electrochemical interface of the working electrode, where the electrochemical double layer recruits and releases charge as the electrostatic potential difference varies with a set amplitude defined by V_{ref}. The induced oscillation of the electrostatic potential causes charges to be recruited to and released from the interface in a cyclic manner, with the frequency of the cycle depending upon the properties of the EDL. In the following, we detail an oscillation cycle for $V_{app} = 0$, with the assumption that the intrinsic difference in electrostatic potential between the electrode and electrolyte is positive

1. We will assume that the depicted cycle begins with $V_{cell} = -V_{ref}$ (**Figure S5a**). Upon reaching this condition, the op-amp flips to a positive output (**Figure S5b**). When the op-amp output is positive, the solution potential increases, attracting negative charges away from the working electrode. This leads to an increase in V_{cell} as the working electrode releases negative charge from the interface (**Figure S5c, Figure S6**).

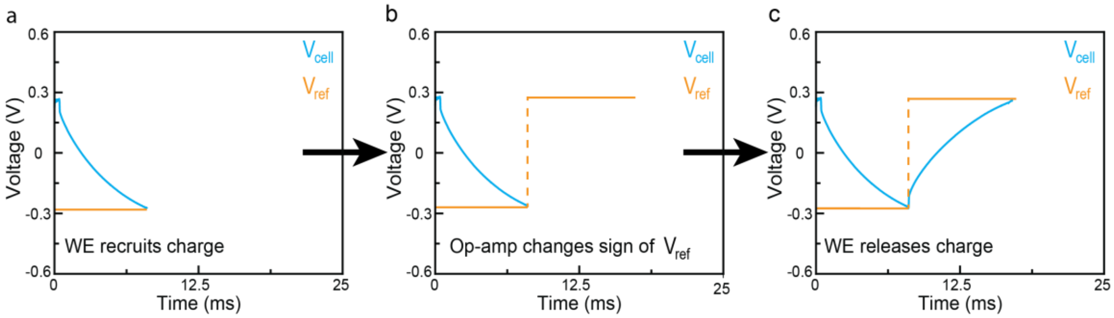

Figure S5. Visual depiction of charge recruit – release cycle showing **(a)** the initial state, **(b)** the change in sign of the op-amp output, and **(c)** the resulting release of negative charges from the electrochemical double layer, leading to an increase of V_{cell}.

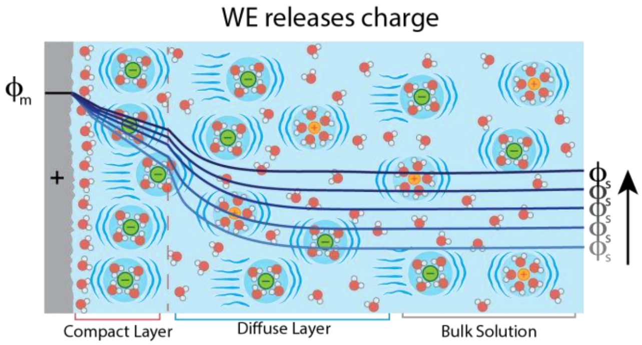

Figure S6. Depiction of EDL behavior when the working electrode is releasing negative charges under condition of positive op-amp output (V_{ref} positive). Illustrations not to scale, rearrangement of solvent molecules and positive charges not drawn for simplicity.

2. Once the voltage across the electrochemical cell equals the positive reference voltage (**Figure S7a**), the operational amplifier once again changes the sign of the output voltage, now becoming negative (**Figure S7b**). When the op-amp output is negative, the solution potential decreases, driving negative charges towards the working electrode. This leads to a decrease in V_{cell} as the working electrode accumulates negative charge at the interface (**Figures S7c, S8**).

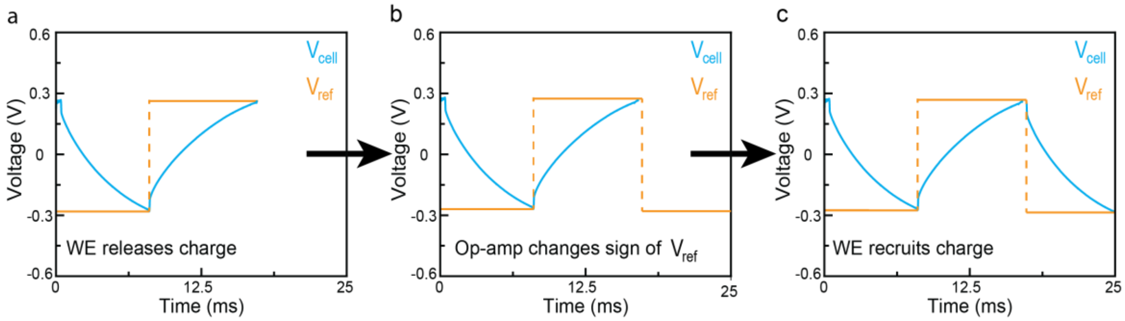

Figure S7. Visual depiction of the charge recruiting phase of the cycle, which starts **(a)** when $V_{cell} = +V_{ref}$ causing **(b)** the change in sign of the op-amp output, and **(c)** the resulting recruitment of negative charges towards the electrochemical double layer, leading to a decrease of V_{cell}.

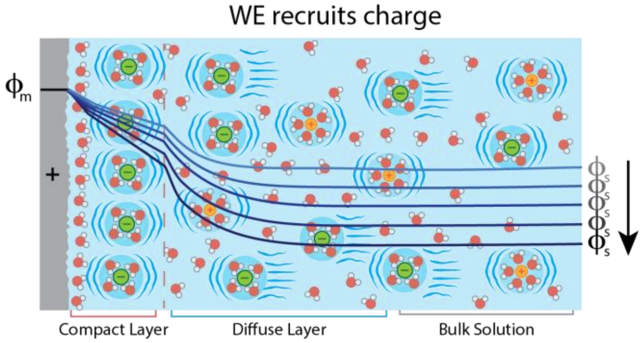

Figure S8. Depiction of EDL behavior when the working electrode is recruiting negative charges to the interface under condition of negative op-amp output (V_{ref} negative). Illustrations not to scale, rearrangement of solvent molecules and positive charges not drawn for simplicity.

3. The charge recruitment and release cycle continuously repeats with the output frequency of the cycle dictated by the time it takes for the interfacial rearrangement of ions and solvent molecules to compensate for the perturbation of interfacial electrostatic potential by the circuit.

4. The same principle applies in the presence of an applied potential. However, in these experiments, we first apply a voltage across the electrochemical cell with a DC power supply to generate a difference in electrostatic potential between the electrode and electrolyte (V_{app}). This leads to the rearrangement of electrolyte components and the modification of the electrochemical double layer as illustrated in **Figure S9**, which changes the time constant of its rearrangement under oscillation and therefore the frequency output of our circuit. The voltage then oscillates between $V_{app}+V_{ref}$ and $V_{app}-V_{ref}$ as described above (**Figure S9**).

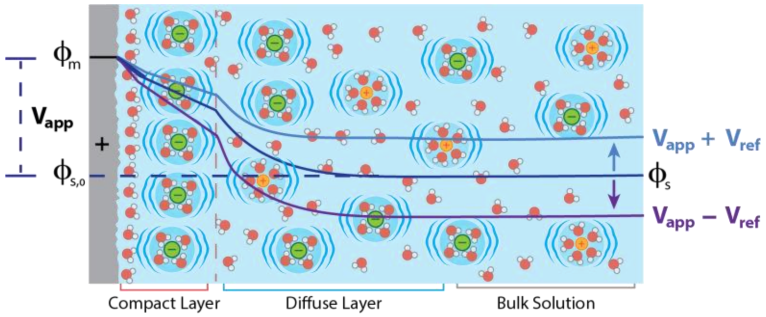

Figure S9. Illustration showing the variation in interfacial potential drop occurring under oscillation in the presence of an applied potential.

Photographs of Setup

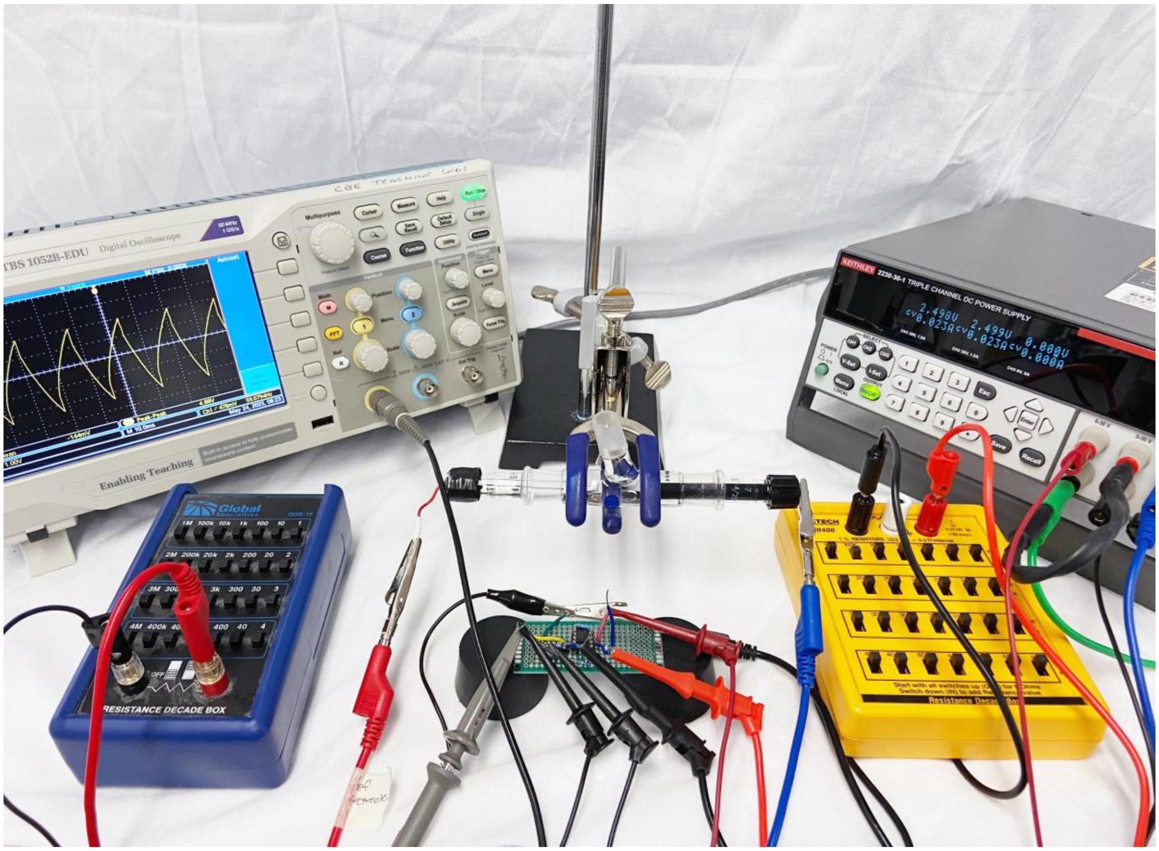

Figure S10. Overall setup with power supply, variable R1 decade box (right), and decade box for solution resistance compensation (left).

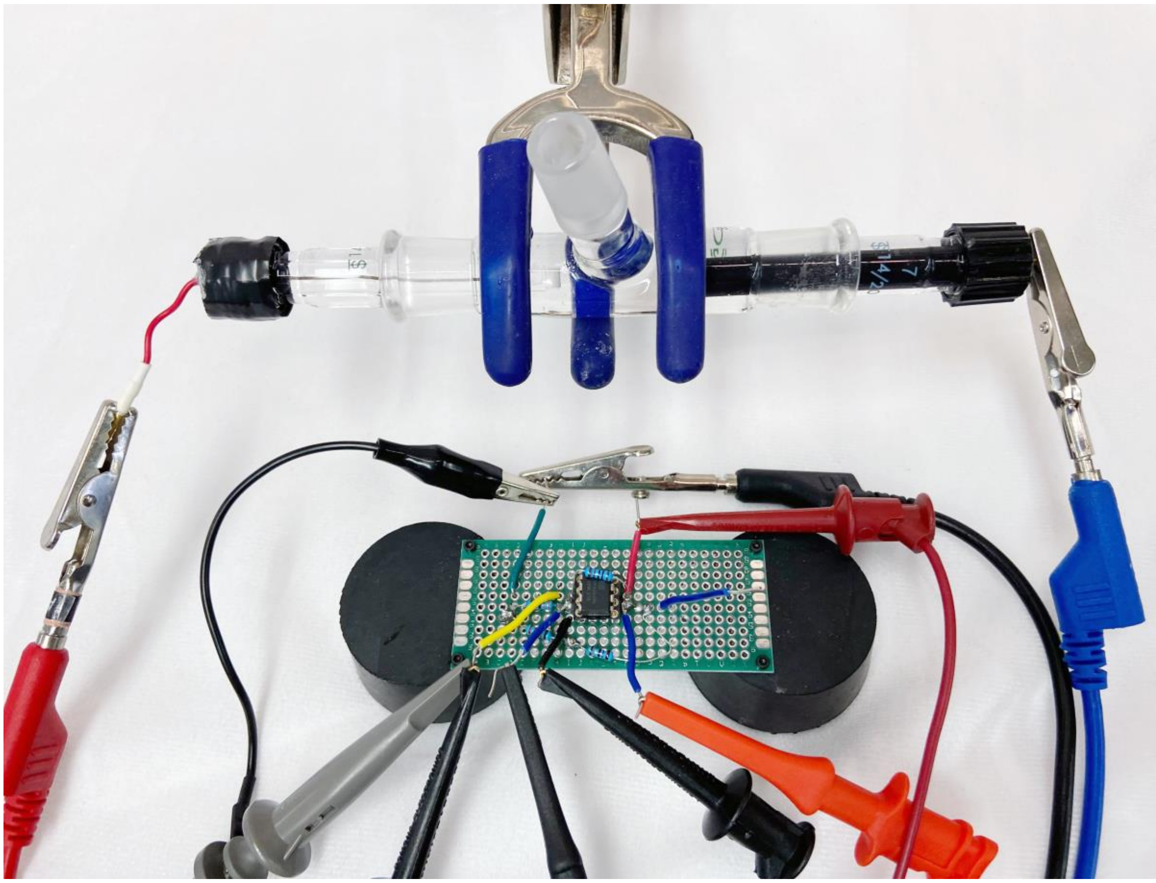
Figure S11. Electrochemical cell and circuit setup.

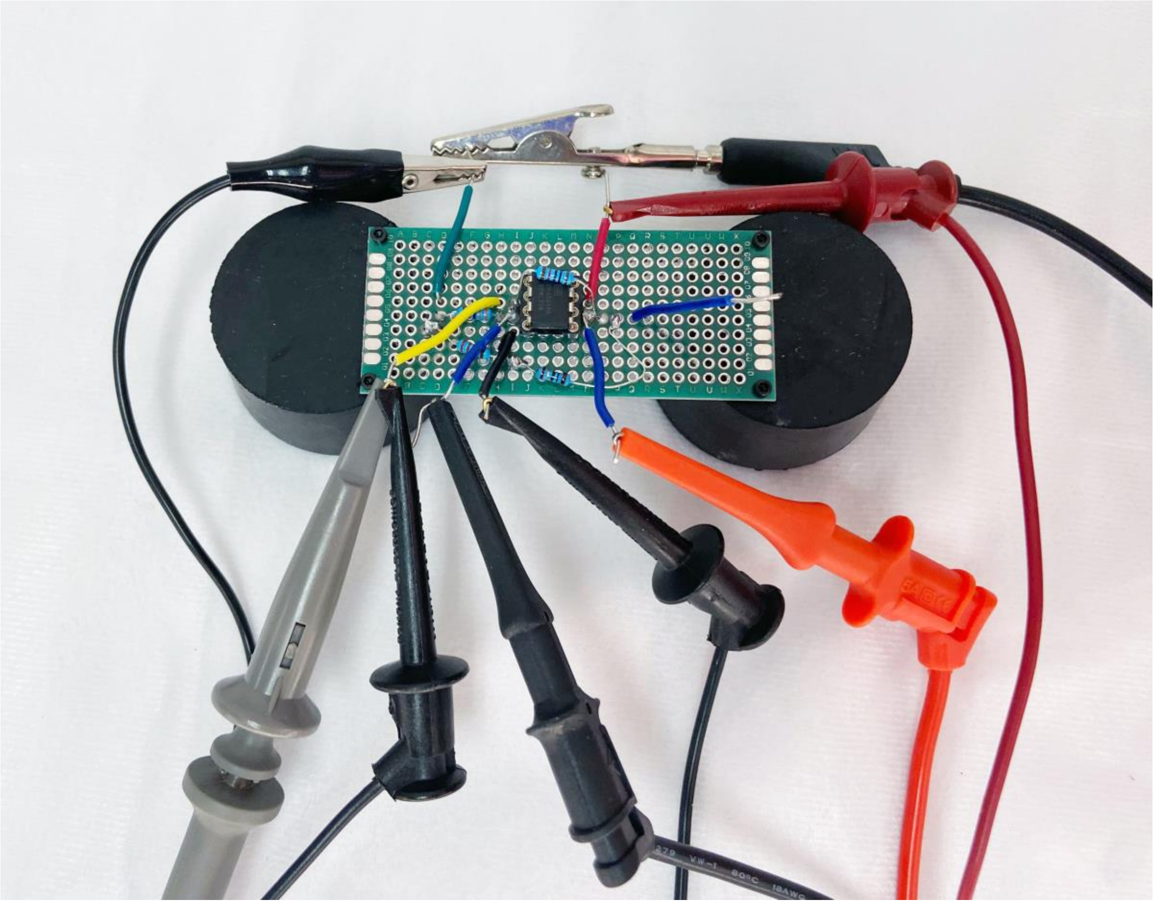

Figure S12. Overview of electric circuit components with oscilloscope probe attached.

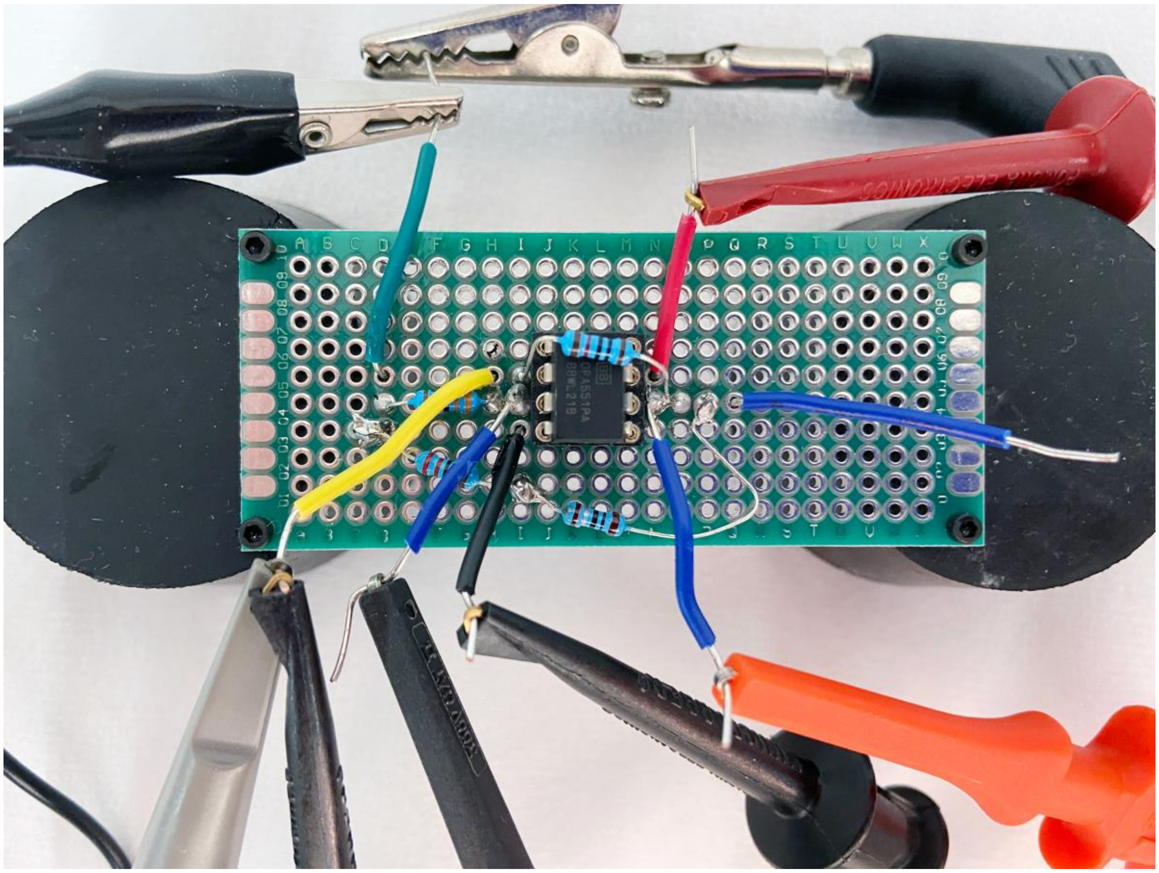

Figure S13. Close-up view of circuit, pin 1 of op-amp is on the upper left.

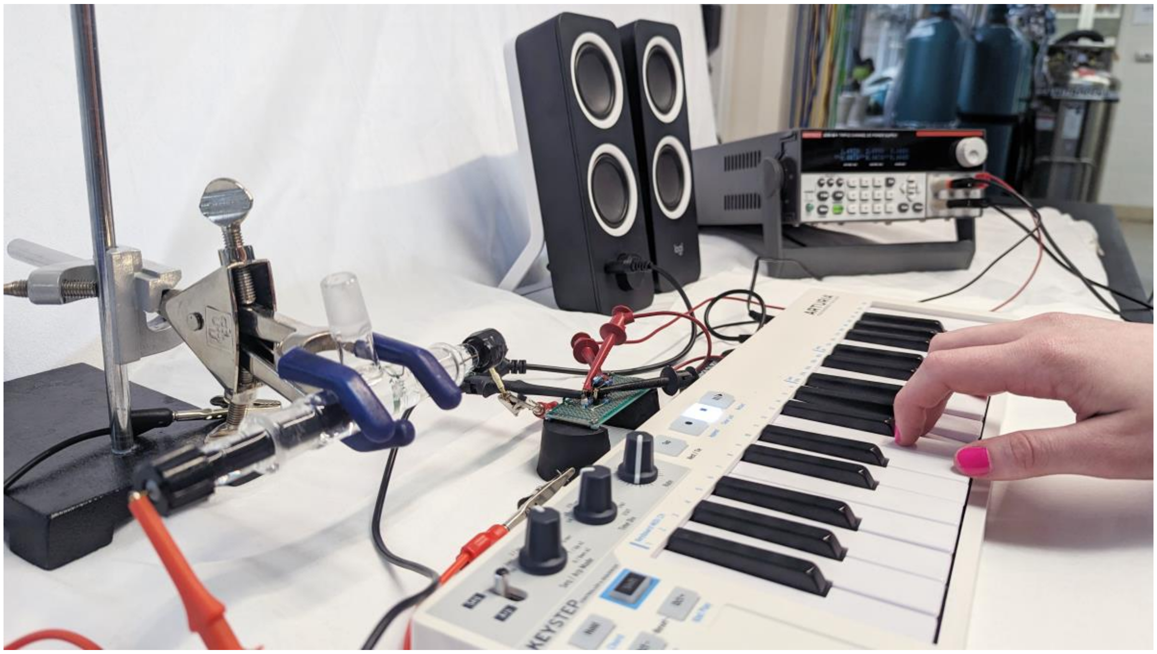

Figure S14. “Electrochemical synthesizer” instrument, using the control voltage output of a keyboard to control V_{app} of the cell.

Impact of Oscillation Amplitude on Frequency Trends

In the concentration study, the peak-to-peak magnitude of the oscillating reference voltage ($V_{pp,ref}$) was 864 mV. To verify whether this amplitude impacts the measured frequency trends, we repeated the experiments on a Pt electrode with a 50 mM $KClO_4$ electrolyte using a peak-to-peak $V_{pp,ref}$ magnitude of 38.4 mV and 312 mV. As shown in **Figure S15**, the potential-dependent trends in frequency were preserved across all oscillation magnitudes. While the observed frequency trends were largely independent of $V_{pp,ref}$, the frequency values increased at lower oscillation magnitudes. This behavior is expected because smaller potential oscillations cause less charge to be recruited and released from the interface.

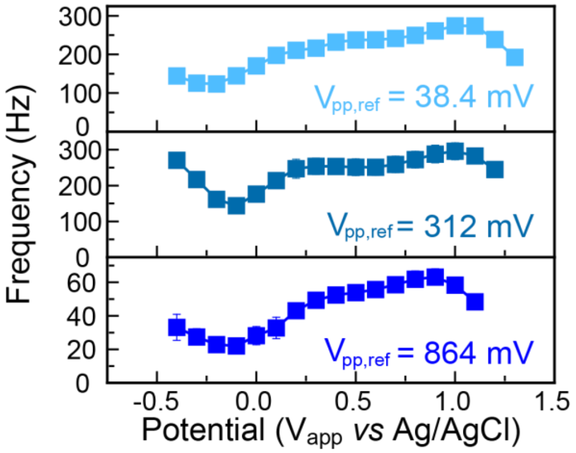

Figure S15. Oscillation frequency for a 1.6 mm OD Pt electrode in 50 mM $KClO_4$ with peak-to-peak oscillation amplitudes of 38.4, 312, and 864 mV. Frequency trends were preserved across different amplitudes.

To conduct these experiments probing the effect of $V_{pp,ref}$, different combinations of R1, R2, and R3 were used (**Table S1**). To reduce $V_{pp,ref}$, the ratio of R1 to R2 was increased by switching R2 from 33 kΩ to 10 kΩ then adjusting R1 as needed. Additionally, R3 was increased to 75 kΩ for the 38.4 mV case to bring the frequency into the audible range. This change in R3 did not impact the frequency trends but contributes to the discrepancy in frequency value measured between the different conditions.

Table S1. Resistor values for the various $V_{pp,ref}$ cases

$V_{pp,ref}$ (mV)	R1 (kΩ)	R2 (kΩ)	R3 (kΩ)
864	33	33	10
312	47	10	10
38.4	470	10	75

Captions for Video Files

Video S1 (separate file). Comparison of audio for 7 mM and 50 mM $KClO_4$ between -0.4 and 1.1 V. 50 mM $KClO_4$ audio was recorded using a National Instruments USB-6001 DAQ and converted to wav files via MATLAB. Audacity was used for sound processing, and Adobe After Effects was used for video compilation.

Video S2 (separate file). Comparison of audio for 1 M Bu_4NCl, Et_4NCl and Pr_4NCl between -0.4 and 1.1 V. Audio was recorded via a Motu-M2 audio amplifier directly into Audacity, and was then stitched together. Adobe After Effects was used for video compilation.

Video S3 (separate file). Video of introduction of 1 M NaCl into 1 M Bu_4NCl at 0 V. Audio was recorded via a Motu-M2 audio amplifier directly into Audacity, and Adobe Premiere was used for combining the audio and video.

Video S4 (separate file). Video of introduction of 1 M Bu4NCl into 1 M NaCl at 0 V. Audio was recorded via a Motu-M2 audio amplifier directly into Audacity, and Adobe Premiere was used for combining the audio and video.

Video S5 (separate file). Video of introduction of 1 M NaCl into 1 M $NaClO_4$ at 0.6 V. Audio was recorded via a Motu-M2 audio amplifier directly into Audacity, and Adobe Premiere was used for combining the audio and video.

Video S6 (separate file). Video of "electrochemical synthesizer" using a Ti electrode in 80 mM $KClO_4$. Audio was recorded via a Motu-M2 audio amplifier directly into Audacity, and Adobe Premiere was used for combining the audio and video.

SI References

1. A. J. Bard, L. R. Faulkner, *Electrochemical Methods Fundamentals and Applications*, 2nd Ed. (John Wiley & Sons, 2001).
